# Influence of Ti-Si-N Nanocomposite Coating on Heat Radiation Resistance of Fireproof Fabrics

**DOI:** 10.3390/ma14133493

**Published:** 2021-06-23

**Authors:** Danuta Miedzińska, Jan Giełżecki, Ryszard Mania, Konstanty Marszalek, Robert Wolański

**Affiliations:** 1 Faculty of Mechanical Engineering, Military University of Technology, Kaliskiego 2 St., 00-908 Warsaw, Poland; 2 Faculty of Production and Power Engineering, University of Agriculture in Krakow, 21 Mickiewicza Ave., 30-059 Krakow, Poland; jan.gielzecki@urk.edu.pl; 3 Advanced Diagnostic Equipment Sp. z o.o., Weissa 7/C1 St., 31-339 Krakow, Poland; rmania@agh.edu.pl; 4 Faculty of Computer Science, AGH University of Science and Technology, Electronics and Telecommunications, 30 Mickiewicza Ave., 30-059 Krakow, Poland; marszale@agh.edu.pl; 5 Institute of Technology, Pedagogical University of Krakow, Podchorążych 2, 30-084 Krakow, Poland; robert.wolanski@up.krakow.pl

**Keywords:** nanocomposite layer, heat flux density, fireproof fabric, magnetron sputtering

## Abstract

Fireproof fabrics are commonly used for protection of fireguards. Such materials must be characterized by improved heat resistance, especially to radiation and flame. In this paper, fireproof fabric (NATAN and PROTON—trademark names) was covered with Ti-Si-N nanocomposite reflective coating using magnetron sputtering. The fabrics were subjected to heat radiation of heat flux density from 0.615 to 2.525 kW/m^2^. A testing stage equipped with a heat source, thermal imaging camera and thermocouples was used. Two variants of the coatings were studied: Ti-Si and (Ti,Si)N considering different thicknesses of layers. The temperature increment and time to reach the pain threshold (60 °C) which corresponds approximately to a 2nd-degree burn according to Henriques criterion were analyzed. In addition, the microstructural analysis of the samples using a scanning electron microscope (SEM) equipped with energy dispersive spectroscopy (EDS) system was performed. The improvement of heat resistance showed for Ti-Si-coated PROTON and NATAN for all tested heat flux densities. Time to reach 60 °C for PROTON fabric increased maximally from 11.23 s (without coating) to 13.13 s (Ti-Si coating) for heat flux density of 0.615 kW/m^2^ and for NATAN—maximally from 7.76 s (without coating) to 11.30 s (Ti-Si coating) for the same heat flux density.

## 1. Introduction

Fireproof fabrics are widely used for constructing fireguards’ clothing. The risks to firefighters are external, and the unpredictable nature of their work requires optimally universal solutions. The body is overloaded due to the presence of a hot or extremely cold, humid or chemically aggressive environment. Each environment has a destructive effect on human body.

The main thermal hazards in the fire environment are [1] thermal radiation, impact of flame, impact of hot elements (slag, metals, glass), interaction with molten metal and glass, contact with hot objects.

A significant threat is the effect of temperature caused by thermal radiation, especially infrared radiation. High temperatures of flame and heated elements of the construction cause high density of heat fluxes affecting the body. These phenomena have a decisive influence on the operating limitations and the development of thermal injuries of rescuers.

The most commonly used protections are insulating and do not fully protect all parts of the body against radiation. The relatively small number of face and head protection with filters is a serious problem. The organs of sight are susceptible even to a slight influence of infrared radiation, because the pupil of the eye does not accommodate to this spectral range.

Personal protection in the form of firefighter-special clothing is insulating. Clothes with reflective effect intended for special fire-fighting actions [2] based on aluminum foils are imperfect constructions and used sporadically. Modern solutions involving the application of layers on materials with the use of vacuum-plasma techniques allow the use of complex protections.

There is a continuous exchange of energy between the environment and the human body. The energy exchange is carried out by [3]: conduction, convection, radiation, evaporation.

The course and nature of these phenomena depend on external factors described by parameters, such as ambient temperature (body contact temperature), temperature of radiating surfaces around the human body, water vapor pressure, and air velocity. The proper functioning of the organism takes place when the internal temperature is stabilized. External thermal loads and the absorption of heat from the surrounding environment determine the disturbance of thermal stabilization. Disturbances in thermal stabilization (thermal equilibrium) of the organism are signaled by an increase or decrease in internal body temperature in relation to the average body temperature (37 ± 0.5 °C) [4].

The external influence on the human body causes the accumulation of heat or its discharge. Incomplete heat exchange triggers physiological reflex responses. Physiological responses are controlled by the nervous system (somatic and autonomic centrifugal channels). Heat production occurs in all tissues of the body, and its discharge and loss to the environment occurs mainly through the skin and, to a much lesser extent, through the respiratory tract. The heat exchange inside the body between heat-producing tissues and other tissues, including the inside of the body and the skin, occurs through tissue conduction and convection through blood [5,6].

The greatest threats to the health and life of rescuers occur in the working conditions of a hot environment, which is standard in fire conditions, in particular in internal (in-house) fires. The hot environment is also called the hot microclimate defined by the WBGT index [°C] (wet bulb globe temperature) [7]. Another parameter that determines the environment of human activity is the PMV (Predicted Mean Vote) thermal comfort index, introduced by Fanger [8]. It is the relationship between the thermal impression expressed in the 7th grade psychophysical rating scale and the variable parameters of the environment. The value of the PMV index for acceptable thermal comfort should be from −0.5 to 0.5 (hot (+3), warm (+2), slightly warm (+1), neutral (0), slightly cool (−1), cool (−2), cold (−3)).

Comprehensive protection of a rescuer includes a set of individual PPE (personal protective equipment) [9,10] forming a multi-layer system. This system [11] is comprised of multi-layer ensembles or, in some cases, separate layers. As part of the comprehensive protection of a rescuer, there are two basic clothing systems. The operation of the first system is based on thermal insulation of the rescuer’s body, and the second on the reflection of energy streams. In the insulating system, the main element of comprehensive protection for a rescuer is special clothing, in which the membrane is covered with a fabric made of carbon (aramid) fibers such as NOMEX, PBI/KEVLAR, and KERMEL [12,13].

**Figure 1:**
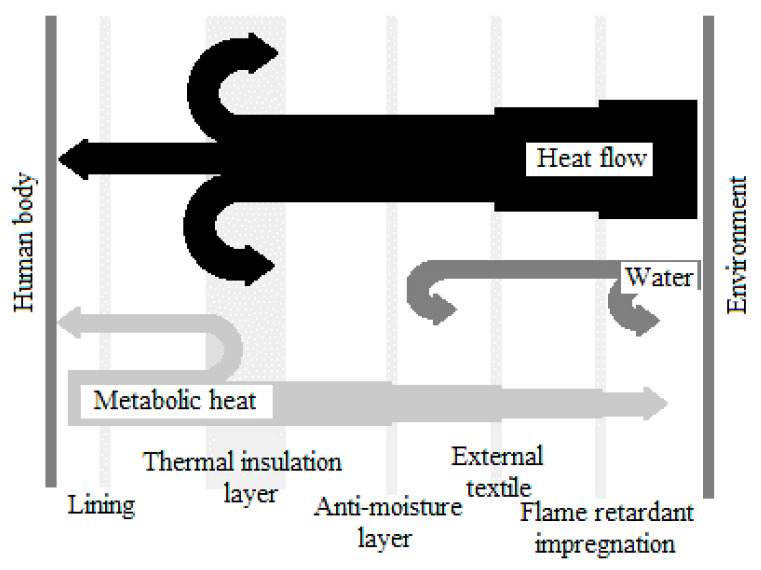
Heat flow during the thermal impact on the rescuer in special firefighter clothing.

The outer material [13] of protective clothing (special clothing, gloves) made of carbon fibers (NOMEX, PBI/KEVLAR, KERMEL) is an insulating barrier, the remaining layers of the set protect against flames and radiation energy streams and moisture. The next layer is thermal and anti-moisture insulation. Thermal insulation is designed to prevent energy streams from entering the skin. The stream of thermal energy from the environment passes through the outer material, the moisture barrier, the insulation layer and the lining of the rescuer’s body (Figure 1). Some solutions take into account the natural role of the underwear used in these cases, and balaclavas in the protection of the head [14].

The basic features of protective materials are: mechanical strength, good thermal insulation, resistance to water, thermal radiation, and good reflection properties.

In a fire with high energy emissions, the implementation of tasks requires, first of all, personal protective equipment with an insulating and reflective effect.

Reflective metallic layers are applied to fabrics intended for the production of protective clothing, mainly by sticking a thin foil [15]. Aluminum foil is commonly used. In addition, the vacuum metallization technology using the vapor deposition process is known, but it is not used on an industrial scale to cover fabrics intended for protective clothing. A literature report describing the process of applying a Zn-Bi coating to a fabric using a magnetron can be found [16]. On the other hand, e-fabrics are modified mainly with semiconducting polymers, such as polyaniline or polypyrrole, and the surface of the fibers is sometimes covered with a thin layer of gold, silver or carbon nanotubes. These are very expensive and complicated technologies, not used on a larger scale [15].

The T-Si-N coatings technology were widely studied in the literature for many years. One of first of such coating analysis was presented by Shizhi at al. in 1992 [17]. The authors studied the morphological properties of Ti-Si-N films prepared by plasma-enhanced chemical vapor deposition. In [18] the authors tested wear and oxidation resistance of TiN–SiN_x_ coating which was prepared using magnetron sputtering from two opposite Ti and Si targets. Kauffmann at al. [19] presented the experimental and numerical hardness analyses of nanocrystalline two-phase TiN/a-SiN_x_ coatings. The authors widen the study with the microstructural size effects in [20].

**Figure 2:**
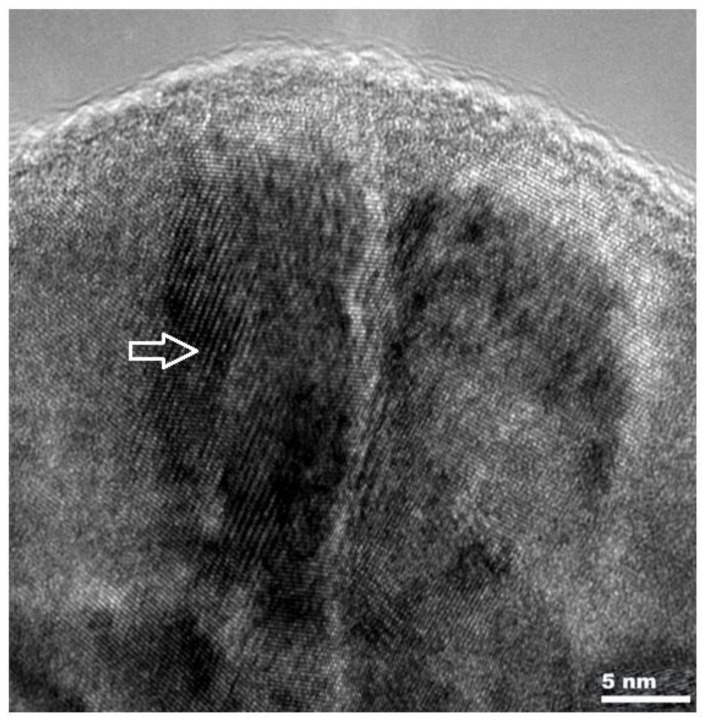
Microstructure of the composite nc-TiN/a-Si_3_N_4_ layer, obtained on a high-resolution electron microscope. White arrow shows TiN nanocrystalic area (ribbed), remaining area is amorphous silicon nitride—Si_3_N_4_.

The research [21,22,23] on composite coatings of titanium nitride—silicon nitride and also chromium nitride—silicon nitride, informed the technology of applying these layers and linking their structure with the conditions in the chamber during the application process. The conducted research allowed for better understanding of the relationship between the observed structure of the layer and its properties. Figure 2 shows a photo from a high-resolution transmission microscope of the nc-TiN/a-Si_3_N_4_ layer deposited using the magnetron sputtering method.

Initial studies, the results of which were partially presented in several publications [22,23,24,25], showed that the temperature of 60 °C—defining the so-called pain threshold, with a heat energy stream with a density of approx. An energy transfer of 36 kW/m^2^ from the source at a temperature of 600 °C, is achieved in the case of a Nomex type fabric within a few to several seconds, and covering this fabric with a layer of, e.g., c-TiN/a-Si_3_N_4_, extends this time from approx. 10 s to approx. 120 s.

Other materials and their modifications that can be found in the literature are, e.g., knitted fabrics with phase change materials designed and produced for use in under-barrier protective clothing [26], polyurethane-coated knitted fabrics [27,28], highly durable polysiloxane-zinc oxide (ZnO) coated polyethylene terephthalate (PET) fabric [29].

The research on the impact of radiation on the materials of personal shields and the assessment of their effectiveness are carried out using many methods. The methods used so far consist in subjecting samples of shielding materials or complete clothing to radiation simulating real conditions and measuring the temperature on the inside of the shields with the use of calorimeters and thermocouples.

**Figure 3:**
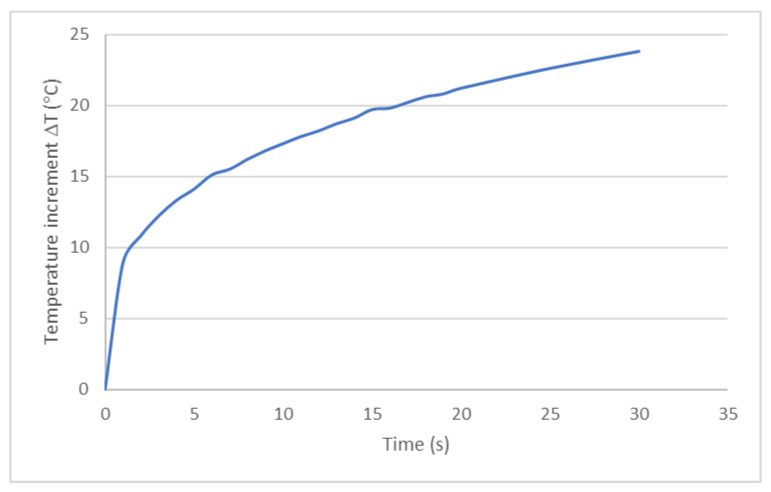
Stoll and Chianta diagram [31].

The research on the effectiveness of protection is most developed in the United States. They are based on several methods. One of them is based on the NFPA 1971–2000 standard (National Fire Protection Association) and the ASTM 4108 standard [30]. The effectiveness evaluation consists in comparing the temperature value after 30 s to the Stoll and Chianta diagram—Figure 3. Time of II stage burn appearance can be assessed as a function of heat flux density range from 4.5 to 50 kW/m^2^ [31,32,33].

NFPA developed also TPP ratio (Thermal Protective Performances) given by the equation [34]:

$$TPP=\tau \cdot I_{t}$$

where: *τ* is a time of radiation action, *I_t_*—heat flux density.

The RPP in the NFPA 1977 test for layer sets of protective clothing for field firefighting must not exceed 290 kW/m^2^.

Other procedures relate to the use of thermal manikins such as THERMOMAN by DuPont or PYROMAN developed by the Center of Research on Protection and Comfort at North Carolina or other [35,36,37,38].

Other methods of heat resistance of textiles assessment are connected with model approach. For example, Zhu and Zhang [39] developed a thermal wave skin model incorporating surface heat flux from a skin to characterize the thermal performance of heat resistant fabrics covering the skin stimulant sensor. Łapka [40] prepared the advanced mathematical and numerical models of energy transfer in the protective clothing and in the superficial tissue.

The aim of this study is to investigate the fabrics used for firefighting clothing—NATAN and PROTON (trademark names) produced by ANDROPOL, covered with a nanocomposite layer, subjected to thermal loads resulting from thermal radiation. These are loads typical for the work of a firefighter. Thanks to the tests presented, it is possible to determine the effectiveness of protection of such fabrics with a modified surface. On the basis of the literature review, it can be concluded that the study is novel in the area the application and testing of Ti-Si-N coating as a reflective barrier to heat radiation.

## 2. Materials

Two types of fireproof fabrics were selected for the study: NATAN and PROTON. Their properties are presented in Table 1.

Those fabrics were covered with the nanocomposite Ti, Si layer:

- Ti-Si—titanium layer with 10% at of silicon,
- (Ti,Si)N—titanium silicon nitride layer, TiN with Si_3_N_4_ admixture.

**Figure 4:**
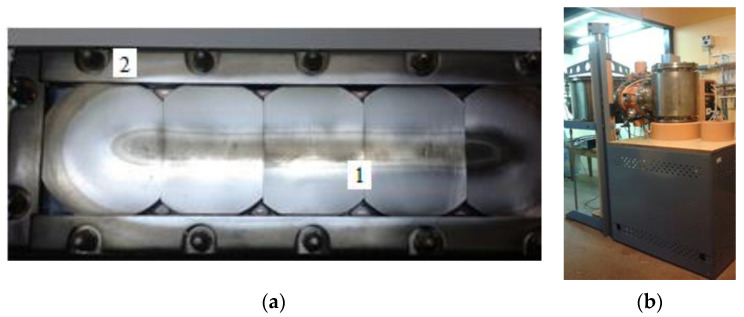
Magnetron sputtering stage: (**a**) linear magnetron (1—Si-Ti sinters, 2—fixtures for fixing sinters); (**b**) vacuum station.

These layers were applied by the PVD method with the use of titanium-silicon sinters with a content of 10% at. Si. Linear magnetron and vacuum station for layering were presented in Figure 4.

**Figure 5:**
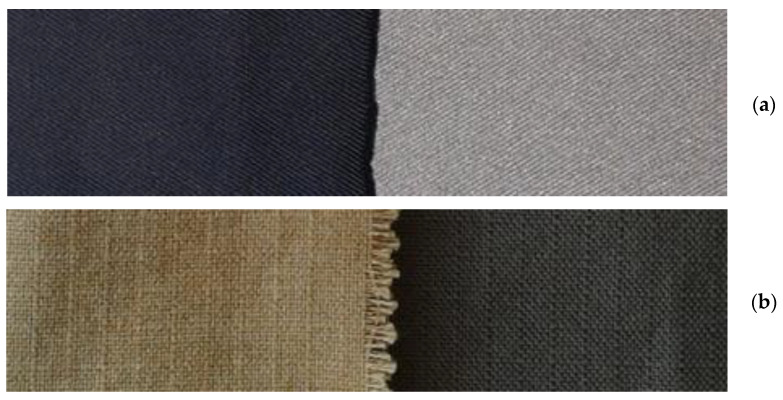
Tested samples: (**a**) PROTON: without coating **(left**), with Ti-Si coating (**right**), (**b**) NATAN: without coating (**left**), with Ti-Si coating (**right**).

Table 2 lists the samples together with the description of the layers and the parameters of their application. Figure 5 shows photos of selected samples. Three samples of each type were tested in the same conditions. The samples were cut from the middle part of the coated fabrics of 1 m^2^ area.

**Figure 6:**
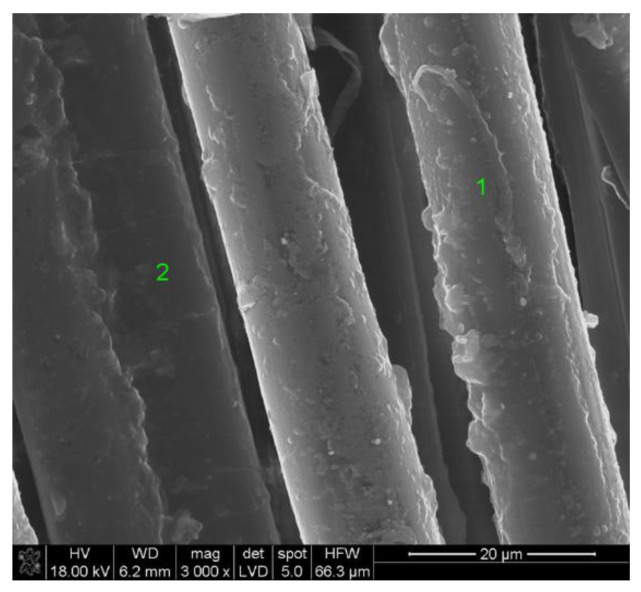
SEM image of (Ti,Si)N coated NATAN fabric, points “1” and “2” are marked for EDS analysis.

**Figure 7:**
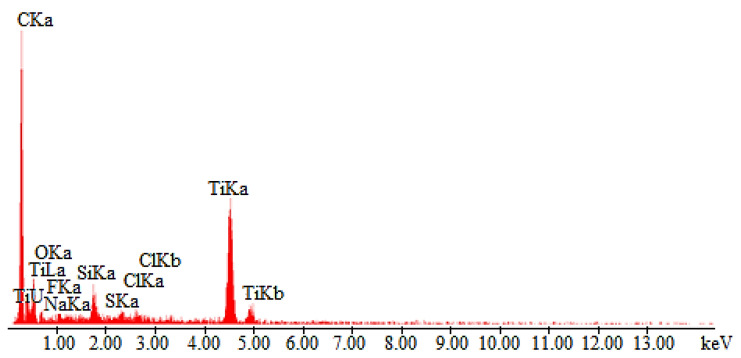
EDS spectrum of element distribution for (Ti,Si)N coated NATAN fabric at point “1” (Figure 6).

The coating microstructural analysis was carried out using a scanning electron microscope (SEM) equipped with energy dispersive spectroscopy (EDS) system. The microscopic image of NATAN fabric fibers covered with a (Ti,Si)N layer was shown in Figure 6, and the analysis of the elemental composition of the layer in point 1 (Figure 6) is shown in Figure 7.

It is worth noting that the tested sample did not require special preparation for observation, despite the fact that the fabric is an electrical insulator and covering it with a layer was a sufficient procedure to discharge the electric charge and make observations in the electron microscope.

**Figure 8:**
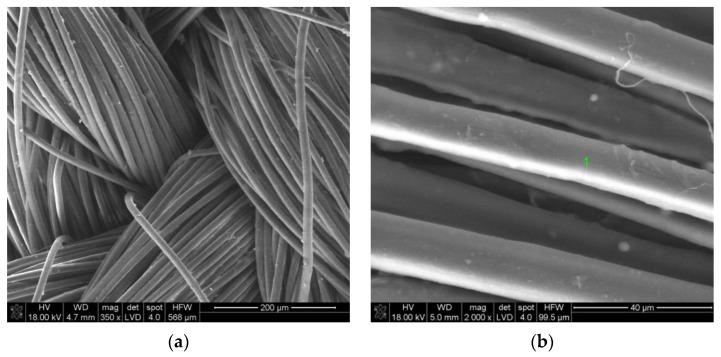
SEM image of Ti-Si coated NATAN fabric at magnification of (**a**) 350×; (**b**) 2000×; point “1” is marked for EDS analysis.

**Figure 9:**
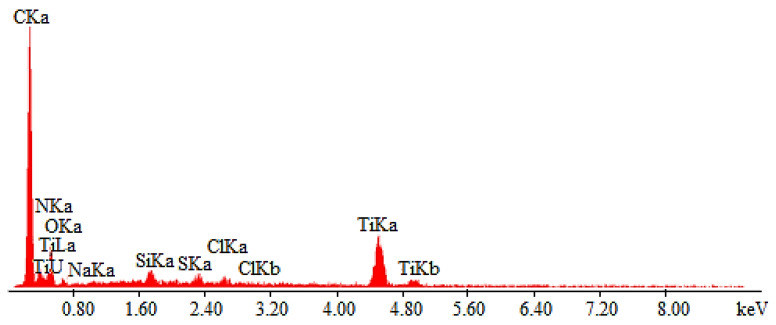
EDS spectrum of element distribution for Ti-Si coated NATAN fabric at point “1” (Figure 8b).

Figure 8a shows a microscopic photo of NATAN fabric covered with a Ti-Si layer. At a low magnification (350×), the weave of the tested fabric is visible. Figure 8b shows the image of the same surface of NATAN fabric obtained at higher magnification (2000×). It can be seen that the layer adheres tightly to the fibers of the fabric. There are visible “bridges” formed by the applied layer in places where the fibers are close to each other. The symbol “1” marks the place where the chemical composition tests were carried out using the EDS method. The result of the performed test is shown in Figure 9.

The presented EDS spectrum shows the chemical composition of the surface (including the base material). The line intensity is proportional to the chemical composition of the analyzed area. Lines are visible for the layer material, titanium, and silicon. Both elements are not present in the fabric. The remaining ones visible in the spectrum come from the fabrics. The height of the Ti and Si lines is proportional to the thickness of the tested layer.

**Figure 10:**
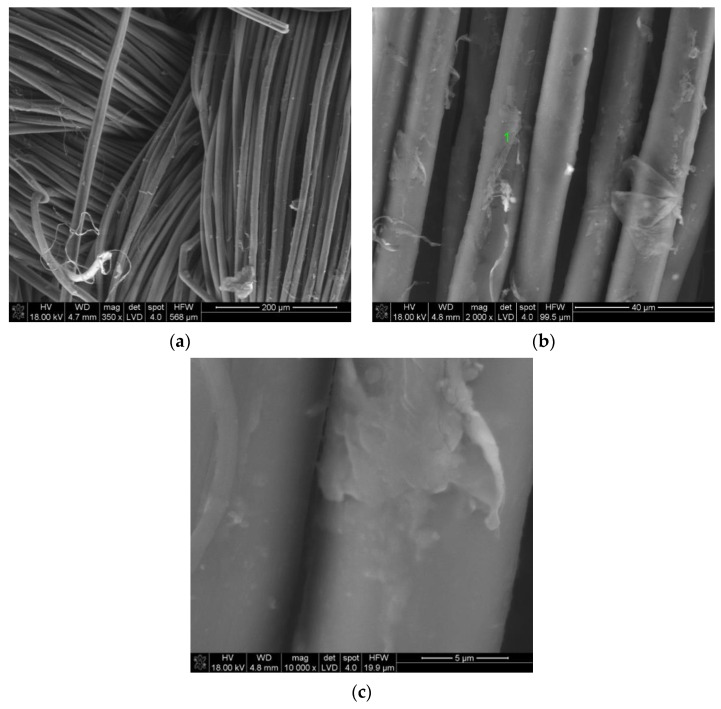
SEM image of Ti-Si coated PROTON fabric at magnification of (**a**) 350×; (**b**) 2000×; (**c**) 10,000×; point “1” is marked for EDS analysis.

The exemplary test results for the PROTON fabric are also presented. Figure 10 shows the microscopic image obtained for the material coated with Ti-Si. Comparing the surface image in Figure 8b and Figure 10b the differences probably caused by different types of substrates can be noticed. The layer on the PROTON fabric does not adhere well to the substrate, which is better shown in Figure 10c.

**Figure 11:**
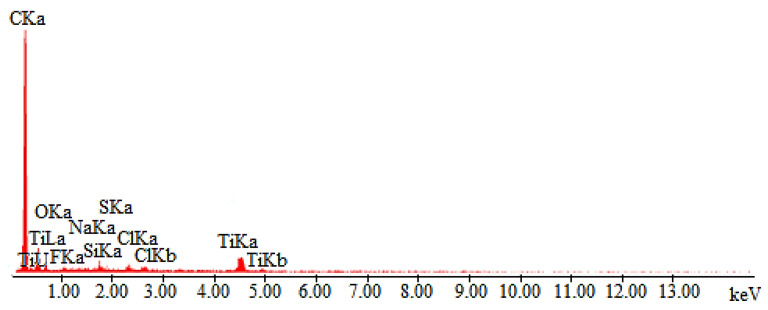
EDS spectrum of element distribution for Ti-Si coated NATAN fabric at point “1” (Figure 10b).

Figure 11 shows the EDS spectrum of the chemical composition the Ti-Si layer on PROTON fabric. Titanium and silicon lines testify to the presence of a Ti-Si layer in the analyzed area. The site selected for analysis is shown in Figure 10b with the number “1”.

## 3. Testing Stage and Methodology

The applied testing stage is used to test the resistance of samples of sets of components or single fabrics used in the construction of protective clothing and those fabrics coated with Ti and Si nanoparticles; Ti-Si/(Ti,Si)N exposed to thermal radiation.

**Figure 12:**
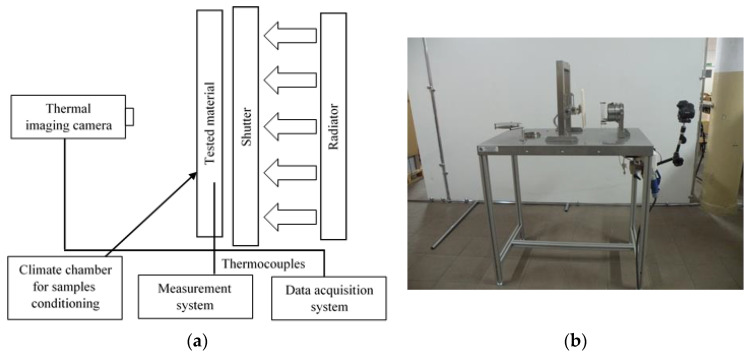
Heat radiation testing stage: (**a**) block scheme; (**b**) real photo.

The stage enables the measurement of the following parameters:

- Time of the heat flux effect on the sample, corresponding to the 2nd-degree burn of the protected body of the rescuer according to the RHTI (radiant heat transfer index) criterion (i.e., based on the specific amount of heat transferred by the sample) [27];
- Time of reaching pain threshold (60 °C criterion) which corresponds approximately to a 2nd-degree burn according to Henriques criterion [27];
- Temperatures during a simulated firefighter body test;
- Temperatures on the surfaces of individual layers during the measurement;
- Heat flux passing through the sample of personal protective equipment and reaching the simulated body of the rescuer during the test;
- The amount of accumulated energy in the sample.
- The stage (Figure 12) consists of:
- Supporting structure with instrumentation, locating the measurement plane at a height convenient for the person conducting the measurement and securing the rest of the elements in space;
- Sliding mechanisms that enable the position of the radiators and their mountings to be changed;
- Radiators—a source of thermal impact;
- A sample—a package of personal protective equipment or a single material;
- Set of sleeve elements tensioning and supporting the sample, fixing the set of sleeve elements of the sample;
- Diffuser (thermal screen) to avoid exposure of the sample to radiation before starting the measurement;
- Climate chamber;
- Stabilizing thermal plate with a temperature similar to that of human skin to simulate the body of a firefighter and used as an auxiliary wall;
- Heat flux sensor—gSKIN^®^-XP (greenTEG AG, Zurich, Switzerland);
- SBG01 heat flux meter (Hukseflux, Delft, The Netherlands);
- K-type thermocouples (Premier Farnell UK Ltd., Leeds, UK);
- Data acquisition system: a computer, NI 9237 bridge analog module (National Instruments, Austin, TX, USA), NI 9214 thermocouple module (National Instruments, Austin, TX, USA), NI cDAQ-9174 (National Instruments, Austin, TX, USA) insulated connection terminal, software dedicated to the stand (Standard Service Program for CompactDAQ Systems, National Instruments, Austin, TX, USA);
- Data acquisition system: Flir T 430sc thermal imaging camera, software dedicated to the station (Flir ResearchIR Max v. 4.40.1.6, Wilsonville, OR, USA);
- Peltier PT-31 ultrathermostat (A Kruss Optronic, Hamburg, Germany).

Infrared heaters with the following properties were selected as the heat source with following properties power supply 230 V AC/50 Hz, smooth temperature/capacity regulation with temperature control through the use of an integrated K-type thermocouple (NiCr-NiAl), maximum temperature 900 °C, infrared emission range from 2 to 10 µm, square characteristics of heat impulse.

The testing stage includes the innovative solutions for equipment elements. Such elements include both solid parts, such as a thermal stabilizing aluminum plate, sleeve elements for tensioning and supporting the sample, and a mounting socket, as well as software with a measurement and data acquisition system and developed criteria for assessing the effectiveness of personal protection. The application of the limit temperature of 60 °C (so-called pain threshold) as a criterion for the risk of second-degree burns, and thus the criterion for assessing the effectiveness of the tested protections is innovative in relation to current standards. At the same time, during measurements, the RHTI parameter currently used in standard tests is determined.

**Figure 13:**
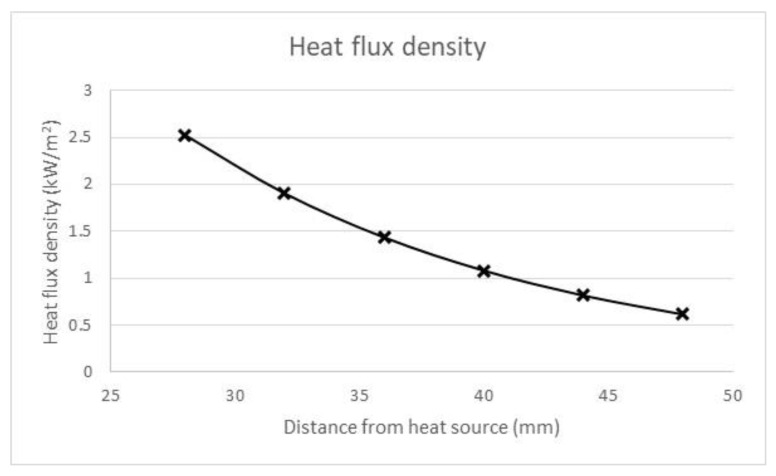
Heat flux density vs. distance between heat source and sample in testing stage.

The samples were irradiated with a heat flux density ranging from 0.615 to 2.525 kW/m^2^. The heat flux density was regulated by changing the distance between the heat source and the sample, according to the diagram shown in Figure 13.

**Figure 14:**
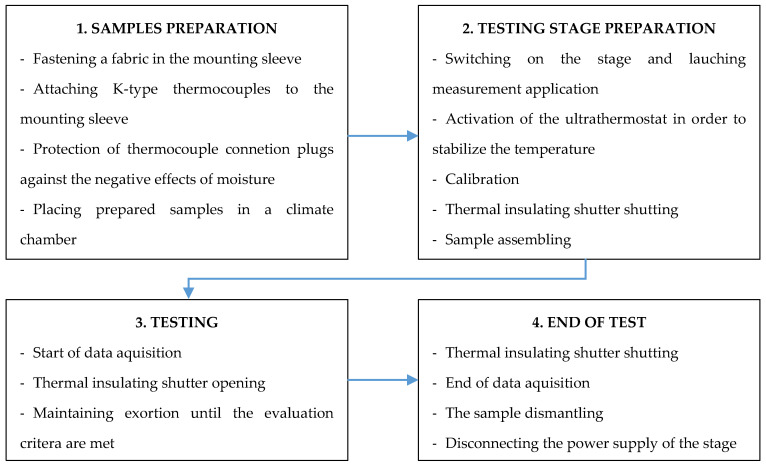
Scheme of testing methodology.

The measurement was carried out using thermocouples and a thermal imaging camera at the same ambient humidity of 65% and ambient temperature of 24 °C. The tests were carried out until the temperature reached 60 °C (pain threshold corresponding to a 2nd-degree burn from Henriques criterion). The schematic diagram of testing methodology was presented in Figure 14.

## 4. Results and Discussion

**Figure 15:**
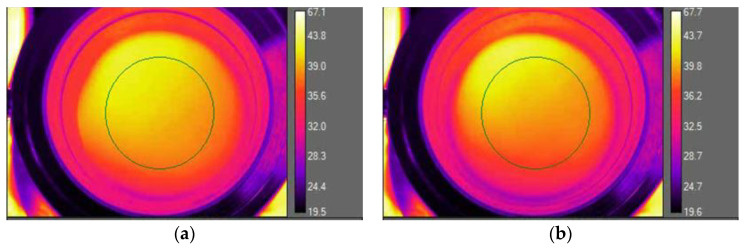
Thermograms of a material sample exposed to thermal radiation: PROTON without coating (**a**) and coated (**b**).

As was mentioned above, the tests were carried out with the use of a thermal imaging camera. The temperature on the inside of the material exposed to thermal radiation was measured with the FLIR T430sc thermal imaging camera (Teledyne FLIR LLC, Wilsonwille, OR, USA). The camera recorded thermograms with a frequency of 30 Hz and saved them on the computer’s hard drive. The results were processed using the Flir ResearchIR Software program to determine the mean temperature of the area of the marked fragment of the sample, the example of which was presented in Figure 15.

**Figure 16:**
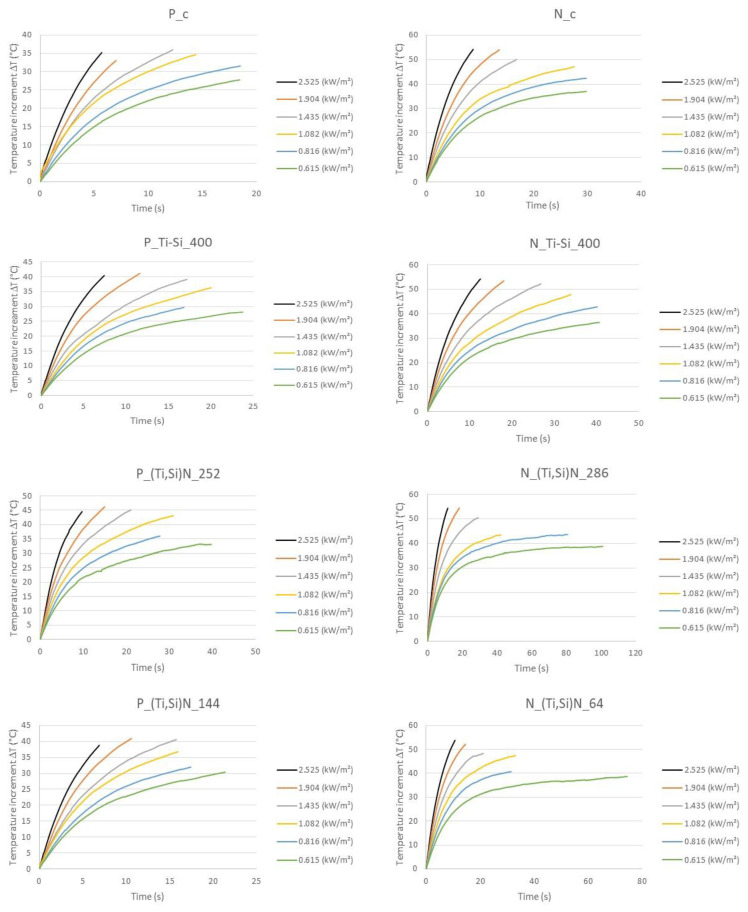
Results of heat radiation tests (samples named in accordance to Table 2).

The relationship of the temperature increment and time for each sample and each heat flux density value was achieved. The results were presented in Figure 16. The results are the average of three tested samples. The results were almost fully repeatable.

In order to assess the influence of the nanocomposite layer on the protective properties of the selected fireproof fabrics, the values of the time for which the inner side of the sample reached the pain threshold temperature (60 °C) were determined.

**Figure 17:**
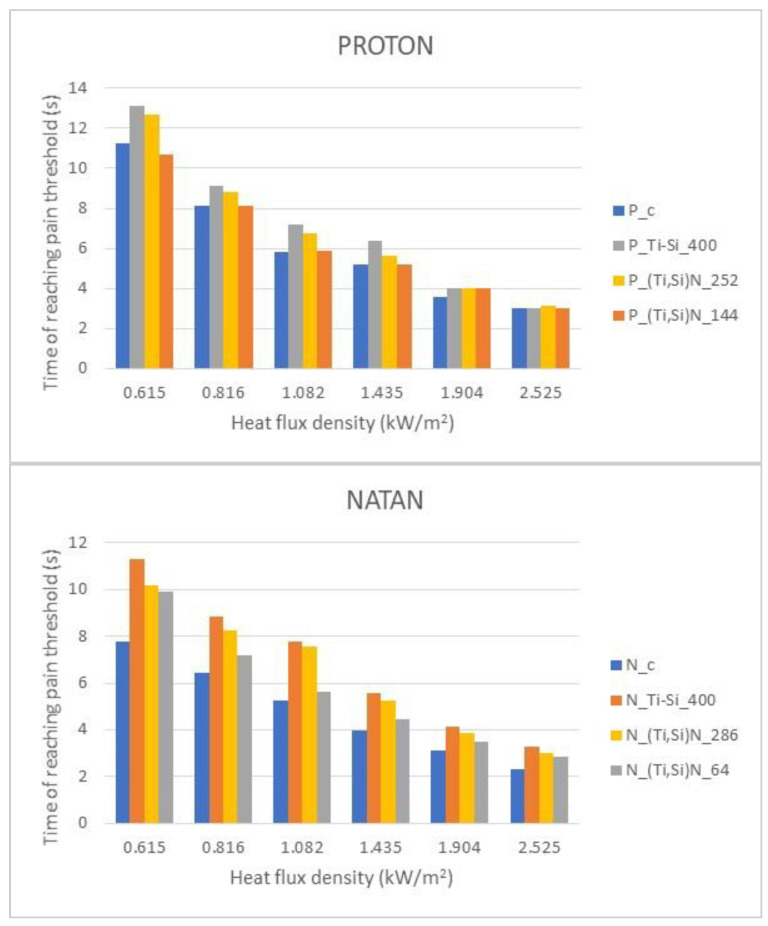
Summary of pain threshold reaching time for NATAN and PROTON samples in accordance to heat flux density.

The results were summarized as bar graphs for NATAN and PROTON and shown in Figure 17. To present the accurate values of the test Table 3 was prepared.

The obtained test results show the influence of the applied nanocomposite reflective layer on the resistance of the special fabric to the effects of thermal radiation.

Data for materials without a layer and with a reflective layer of three types: Ti-Si with a thickness of 400 nm and (Ti,Si)N with two thicknesses (286 and 64 nm for NATAN and 252 and 144 for PROTON) were analyzed. The tested samples were exposed to thermal radiation with a flux density of 0.615, 0.816, 1.082, 1.904, and 2.525 kW/m^2^.

The time of reaching the temperature of the conventional pain threshold (60 °C) on the inside of the fabric was determined as the parameter used to compare the effectiveness of the fabric protection against thermal radiation.

The results presented in Figure 17 allow to conclude that in practically every case studied, the nanocomposite layer increases the heat resistance of the fabric covered with it, and the intensity of improvement is the most favorable for the lower values of the heat flux density. On the base of values presented in Table 3, it can be shown that time of reaching 60 °C for PROTON fabric increased maximally from 11.23 s (without coating) to 13.13 s (Ti-Si coating) for heat flux density of 0.615 kW/m^2^ and for NATAN—maximally from 7.76 s (without coating) to 11.30 s (Ti-Si coating) for the same heat flux density.

## 5. Conclusions

**Figure 18:**
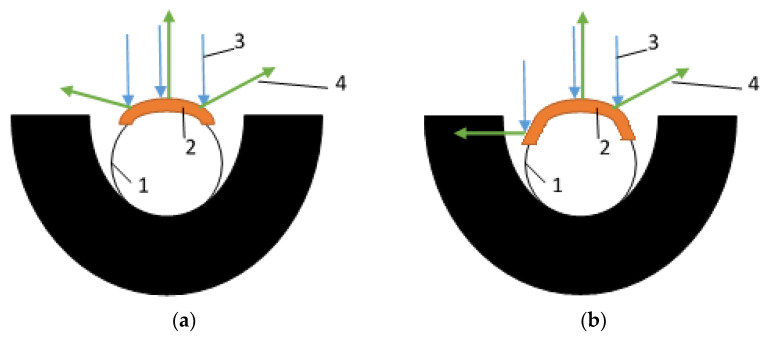
Mechanism of heat radiation reflection in fabric: (**a**) with coated top of fibre; (**b**) with coated top and side walls of fibre; 1—fibre, 2—coating, 3—incident radiation, 4—reflected radiation.

The 400 nm thick Ti-Si layer (without N inclusions and applied as a single layer) was the most effective for both NATAN and PROTON fabrics. This effect may be due to the quality of the applied layer resulting from the application process. In the case of (Ti,Si)N layers, these layers were applied in two or three stages, and the base was a Ti-Si layer to ensure high adhesion of the layer to the fabric (which is important for use in the form of clothing that is subjected to, e.g., washing processes)—Table 2. Such process may cause the layer to be applied not only to the upper surface of the fabric, but also to the side walls of the fibers (which can be seen in Figure 6, Figure 8 and Figure 10). This may result in reflection of the radiation at an angle and its return to the fabric, as schematically shown in Figure 18.

Additionally, it should be noted that a better effect of improving heat resistance was observed for the NATAN fabric, which may be the result of a different, flatter weave of fibers in this fabric (Figure 5) and observing the EDS analysis results.

Finally, it can be concluded that the nanocomposite reflective layer can positively affect the heat resistance of the protective fabric used for clothing for firefighters. It should be noted, however, that the selection of the type and composition of the layer as well as the process of its application must take into account both the protective properties and operating conditions (expected heat flux density and its duration), as well as other parameters, e.g., layer adhesion to the fabric and the change in its specific weight.

The presented work will be developed in the future. The authors plan the tests of flame and hot elements impact, improvement of the layering process and finally the numerical modeling of presented thermal effects.

## Figures and Tables

**Table 1:** NATAN and PROTON fabrics characteristics.

Composition	NATAN		PROTON	
Content, %	Nomex^®^ 1,7 dtex	75	PBI	40
Content, %	Kevlar	23	Paraaramid	58
Content, %	P140	2	antystatic	2
Surface density, g/m^2^	195		195	

**Table 2:** Layering parameters of tested samples.

Sample Symbol	P_c	N_c	P_Ti-Si_400	N_Ti-Si_400	P_(Ti,Si)N_252	N_(Ti,Si)N_286	P_(Ti,Si)N_144	N_(Ti,Si)N_64
Base material	PROTON	NATAN	PROTON	NATAN	PROTON	NATAN	PROTON	NATAN
Layer composition	No layer	No layer	TiSi	TiSi	Ti,Si 18 nm + (Ti,Si)N 21 nm × 6 + Ti,Si 18 nm	Ti,Si 18 nm + (Ti,Si)N 22 nm × 5 + Ti,Si 18 nm	Ti,Si 30 nm + (Ti,Si)N 114 nm	Ti,Si 19 nm + (Ti,Si)N 45 nm
Layer thickness [nm]	-	-	400	400	252	286	144	64
Layering parameters								
Partial pressures of gases [Pa]	-	-	P_Ar_ = 0.116 P_N2_ = 0.217	P_Ar_ = 0.116 P_N2_ = 0.217	P_Ar_ = 0.18 P_N2_ = 0.06	P_Ar_ = 0.18 P_N2_ = 0.12	P_Ar_ = 0.116 P_N2_ = 0.101	P_Ar_ = 0.15 P_N2_ = 0.10
Total pressure P_Ar_ + P_N2_ [Pa]	-	-	0.333	0.333	0.24	0.30	0.217	0.25
Electric current intensity M1/M2* [A]	-	-	1.14/1.07	1.14/1.07	1.50/1.50	1.05/1.05	1.14/1.07	1.03/1.05
Effective power M1/M2 [kW]	-	-	0.87/0.71	0.87/0.71	1.02/0.93	0.80/0.71	0.87/0.71	0.87/0.77
Time of layering	-	-	2 min in Ar + 20 min in Ar+N_2_ + 55 min in Ar = 57 min	2 min in Ar + 20 min in Ar +N_2_ + 55 min in Ar = 57 min	1.5 min in Ar + 50 min w Ar + N_2_ = 51.5 min	2 min in Ar + 20 min in Ar +N_2_ + 2 min w Ar = 24 min	1.5 min in Ar +19.5 min in Ar + N_2_ = 21 min	1 min in Ar + 9 min in Ar + N_2_ = 10 min
End temperature [°C]	-	-	196	196	140	97	196	140

**Table 3:** Pain threshold reaching time for NATAN and PROTON samples in accordance to heat flux density.

Pain Threshold Reaching Time (s)							
Heat Flux Density (kW/m^2^)		0.615	0.816	1.082	1.435	1.904	2.525
Sample	P_c	11.23	8.13	5.80	5.20	3.60	3.00
	P_Ti-Si_400	13.13	9.10	7.20	6.37	4.00	3.03
	P_(Ti,Si)N_252	12.70	8.80	6.76	5.60	4.03	3.10
	P_(Ti,Si)N_144	10.70	8.10	5.87	5.16	4.00	3.03
	N_c	7.76	6.43	5.23	3.96	3.10	2.30
	N_Ti-Si_400	11.30	8.87	7.77	5.57	4.13	3.26
	N_(Ti,Si)N_286	10.20	8.26	7.53	5.26	3.87	3.00
	N_(Ti,Si)N_64	9.90	7.16	5.63	4.43	3.47	2.83

## Data Availability

Data available after contact with authors, not publicly archived.
